# Beliefs about food allergies in adolescents aged 11–19 years: A systematic review

**DOI:** 10.1002/clt2.12142

**Published:** 2022-04-06

**Authors:** Kristina L. Newman, Angel Chater, Rebecca C. Knibb

**Affiliations:** ^1^ Psychology Department School of Life and Health Sciences Aston University Birmingham UK; ^2^ Department of Psychology School of Social Sciences Nottingham Trent University Nottingham UK; ^3^ Department for Sport Science and Physical Activity University of Bedfordshire Bedford UK

**Keywords:** adolescents, anaphylaxis, beliefs, children, food allergy

## Abstract

**Aims:**

Research suggests of people with food allergy (FA), adolescents have the highest risk of fatal allergic reactions to food, yet understanding of this population and how they manage their condition is limited. Understanding beliefs and how they affect behaviour could inform ways to reduce risk taking behaviour and fatal reactions in adolescents. This systematic review aimed to explore beliefs adolescents hold about their FA, and how these may be associated with FA management.

**Demographics:**

Adolescents aged 11–19 years with FA.

**Methodology:**

A systematic search of seven databases was conducted. Papers of any design were included that reported on the beliefs about FA in adolescents aged 11–19 years. Data was systemised by narrative thematic analysis.

**Findings:**

20 studies were included. Themes included navigating FA in different environments, carriage and use of adrenaline auto‐injectors, management of the risk of anaphylaxis, behaviour and understanding of others, and food‐allergic identity.

**Implications:**

Adolescents with FA hold a variety of condition beliefs; some beliefs were related to behaviour that could lead to an allergic reaction, while other beliefs were related to protective behaviours. Further research into understanding adolescent beliefs in order to inform clinical management and reduce the risk of potential fatal reactions is essential.

## 1 INTRODUCTION

Food allergy (FA) is classified as an adverse reaction to ingestion of certain foods, with prevalence of FA as high as 10%.^1^ Adolescents and young people with FA are the age group that has the highest frequency of fatal reactions due to serious allergic reactions.^2^ In the European Anaphylaxis Registry, a total of 3514 cases of food‐induced anaphylaxis were reported between July 2007 and March 2018, 56% in patients under 18 years of age, and peanut anaphylaxis was recorded in 459 children and adolescents (85% of all peanut anaphylaxis cases), with most cases labelled as ‘severe’.^3^ Potential causes of the figures for this age group may be identified from Sampson et al.’s^4^ report that 54% of their sample aged 13–21 years purposely ate potentially unsafe foods while 29% did not always carry their adrenaline auto injector (AAI) the only medication for anaphylaxis, a life‐threatening severe allergic reaction. Adolescence is therefore a critical period to examine in those with FA, to attempt to reduce the likelihood of these reactions.

The impact of FA and how it is managed is thought to change as children develop, with adolescence as a time of transition where their beliefs about themselves, their world and their condition may change. Up to the age of 8 years, children tend to rely on parents to manage their FA, and after this age as they progress into adolescence, they become more aware of the difficulties of managing FA and believe it is more dangerous, leading to anxiety.^5^ Over the age of 12 years, children have been reported to experience greater conflict with their parents regarding their FA^5^ as beliefs begin to misalign (e.g., that parents know best and can always keep them safe) which may lead to risky behaviour as adolescents try to assert their independence.

Condition beliefs, how the adolescents feel about their FA, may provide insight into why they engage in these behaviours, but research of this topic is very limited. Beliefs are important as they may directly inform protective behaviours such as AAI carriage and label‐checking, which may reduce reactions.^6^, ^7^ Qualitative research has highlighted adolescents hold strong beliefs about their AAIs, expressing the belief that AAIs are inconvenient due to their bulk^8^, ^9^ and some adolescents are afraid to use them due to the needle injection.^10^, ^11^ Beliefs of peers are also important for adolescents as these may influence the way in which children with FA manage their condition.^12^, ^13^ A review on adolescent experiences of food‐induced anaphylaxis found beliefs around the adolescents' identity with FA, balance and controlling the uncontrollable to be themes considered to directly influence the adolescents' experiences.^14^

There are currently no systematic reviews on adolescent beliefs about their FA. This systematic review, therefore, aimed to explore adolescent beliefs about their FA and how these may be associated with FA management behaviours.

## 2 METHODS

This review is reported using the updated 2020 Preferred Reporting Items for Systematic Reviews and Meta‐Analyses (PRISMA) guidelines^15^ and the protocol was registered on PROSPERO (registration number: CRD42019133576).

### 2.1 Eligibility criteria

The eligibility criteria and search terms were developed using the SPIDER (Sample, Phenomenon of Interest, Design, Evaluation, Research type) tool.^16^ To be eligible, peer‐reviewed articles were required to have sampled participants within the age range of 11–19 years with FA, explicitly discuss or contain significant analysis relevant to beliefs about FA, and be written in English. If papers also included participants outside of this age range but were explicit via quotes or written clarification as to what age the results referred to, these were included. Data from participants outside the age range was not included in analysis. Papers were considered if published between the years 2000 and 2022, as according to the UK's National Health Service, FA has ‘risen sharply’ during this time.^17^ The Mixed Methods Appraisal Tool (MMAT)^18^ was used to assess the quality of studies to be included, and studies were excluded if they were assessed as poor quality. Eligibility criteria are outlined in Table 1.

**TABLE 1:** Sample, Phenomenon of Interest, Design, Evaluation, Research type (SPIDER) tool

Category	Inclusion criteria	Exclusion criteria
Sample	Aged 11–19 years with a FA	Aged outside of 11–19 years or without FA
Phenomenon of interest	Beliefs of adolescents with FA	Not explicitly beliefs (e.g., quality of life) or with no mention of FA
Design	Qualitative, quantitative or mixed methods papers	Abstract, presentation, non‐academic articles or reviews
Evaluation	Evaluation of title and abstract, and then full paper if potentially relevant (including related terms such as ‘belief’, ‘attitude’)	No relevant data in main sections
Research type	Qualitative, quantitative, or mixed methods	None

Abbreviation: FA, food allergy.

### 2.2 Search procedure

A systematic literature search was conducted by KN using seven electronic databases: Cochrane Library, ProQuest, PubMed, Science Direct, Scopus, Web of Science and Wiley, with the final search completed in January 2022. Additional papers were also searched through reference chaining, which involved systematically searching through references of reviewed articles for papers of relevance or interest to the topic to see if eligible papers were missed in the original database searches.

### 2.3 Study selection

Records were assessed for eligibility by KN and RK and duplicates were removed by KN. Article titles and abstracts were read and removed by KN and RK if they did not meet eligibility criteria. Following analysis of abstracts, relevant papers were read in full. Full papers were reviewed by KN and RK and excluded if they did not meet the eligibility criteria.

### 2.4 Synthesis of results

Adolescent FA beliefs were extracted from the study results and reported. As both qualitative and quantitative data was analysed, and a variety of designs and outcomes emerged, a narrative synthesis incorporating thematic analysis^19^ was used to communicate the results. Subgroup analysis was also conducted to assess differences between study samples, settings, and methods.

## 3 RESULTS

**FIGURE 1:**
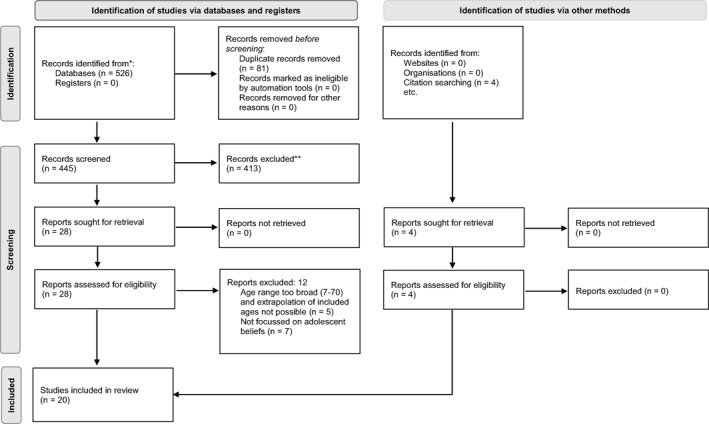
Preferred Reporting Items for Systematic Reviews and Meta‐Analyses (PRISMA) flow diagram of systematic search into adolescent food allergy beliefs

A total of 526 research papers were identified during the database search, with 4 additional papers found through reference chaining, 81 duplicates were removed to leave 449 papers. Following abstract review, 32 papers were read in full, and 12 papers were excluded as they did not meet inclusion criteria. A total of 20 studies met eligibility criteria and were included in data extraction (Figure 1).

### 3.1 Study characteristics

A total of 16 studies used qualitative methods and four used quantitative methods. Papers reported data on approximately 956 participants aged 6–21 years; however, results reported for participants outside the age range for this review were excluded from analysis. Studies were conducted in Canada (*N* = 3), Ireland (*N* = 1), the Netherlands (*N* = 1), Denmark (*N* = 1), Sweden (*N* = 2), United Kingdom (*N* = 11) and across Europe (*N* = 1). Included papers were published from 2007 to 2020. Interviews and focus groups were the main methods used, while the quantitative studies used questionnaires. Table 2 provides details for each included study.

**TABLE 2:** Study characteristics

Authors	Location	Participants	Method	Outcomes
Akeson et al. (2007)	South of Scotland, UK.	7 adolescents (13–16 years), with clinician‐diagnosed anaphylaxis and 8 parents.	Semi‐structured interviews analysed using the framework approach.	Themes: Allergy perceived as ‘not a big deal’; mostly respect for and confidence in managing the allergy but less knowledgeable than parents; mistrust in food labelling; lower and narrower perception of risk in comparison with parents; inconsistency in carrying adrenaline due to practical and psychosocial obstacles
Dean et al. (2016)	Ontario, Canada	10 children (8–12 years), 10 youth (13–17 years), with food allergy and at risk of anaphylaxis.	Semi‐structured interviews analysed using thematic analysis based on grounded theory.	Main theme: Health‐related stigma. Sub‐themes: Disclosure; stigmatisation; normalisation; tension and disclosure.
DunnGalvin et al. (2009)	Cork, Ireland	62 children (6–15 years), issued with an AAI.	15 focus groups, analysed using grounded theory.	Themes: Meanings of food; peer relationships; autonomy, control and self‐efficacy; risk and safety; self/identity; coping strategies.
DunnGalvin et al. (2020)	United Kingdom, France, Germany, Ireland, Spain, Italy, Denmark and the Netherlands	107 participants were interviewed: 24 children, 39 teenagers and 44 caregivers with moderate‐severe peanut allergy	Semi‐structured interviews analysed with thematic analysis.	Two conceptual models with themes related to coping and control, driven by the fear of PA reactions, and the associated emotional, social, relationship and work impacts. Factors moderating these impacts included social attitudes and support, child‐caregiver relationship and coping strategies used.
Fenton et al. (2011)	Canada	10 children (8–12 years) and 10 adolescents (13–18 years) with ‘clinical diagnosis of life‐threatening FA.’	Interviews and illustrations/narrative descriptions analysed with thematic analysis, reflective analysis and depth analysis.	Themes: Social and environmental barriers to safety; emotional burden of responsibility; coping strategies; balance of responsibility (transitions); redefining ‘normal’.
Fenton et al. (2013)	Ontario, Canada	10 children (8–12 years) and 10 adolescents (13–17 years) with ‘anaphylactic allergy.’	Interviews and illustrations/narrative descriptions analysed with thematic analysis, reflective analysis and depth analysis.	Themes: Socio‐material spaces; exclusionary spaces; transitioning spaces.
Gallagher et al. (2011)	Scotland, UK	26 adolescents (13–19 years) with ‘history of anaphylaxis.’	Interviews, 8 adolescents and 10 parents took part in the focus groups. Data was thematically coded.	Themes: Carrying and storing auto‐injectors; training in auto‐injector technique; identifying an anaphylactic reaction; administration technique; knowing when to use an auto‐injector; potential interventions to improve epinephrine auto‐injector use among adolescents.
Gallagher et al. (2012)	Scotland, UK	26 adolescents (13–19 years) ‘at risk of anaphylaxis.’	Interviews, data was thematically coded.	Themes: Experiences of anaphylaxis; managing allergies and preventing further reactions; eating away from home; risk and ‘may contain’ labels; support from healthcare professionals; transition from parental to self‐management
James et al. (2020)	London, UK	100 adolescents (11–18 years) ‘with allergy’ and 82% with FA.	BIPQ was used as a measure, while data analysis included non‐parametric analysis and Mann‐Whitney analysis.	‘Time‐line’ (illness duration) followed by ‘concern’, had highest overall scores on the BIPQ suggesting patients are most concerned about the chronic duration of their condition. Females had higher emotional representation and older participants (14–18 years) had stronger beliefs their condition will be long‐lived.
Jones et al. (2014)	South‐East England, UK	188 adolescents (13–19 years) with ‘diagnosis of severe FA and prescription of an AAI.’	Questionnaire developed from HBM & CS‐SRM analysed using factor analysis multiple regression.	Health beliefs, specifically perceived severity and barriers accounted for 21% of the variance in adherence behaviours. CS‐SRM constructs, illness identity, timeline cyclical beliefs and emotional representations explained 25% of the variance.
Jones et al. (2015)	South‐East England, UK	188 adolescents (13–19 years) with ‘hospital prescribed AAI for FA.’	Questionnaire scale developed from HBM. Logistic regression was used in analysis.	Adherence was more likely if adolescents believed their FA was severe and perceived fewer barriers to disease management. Belonging to a support group and having an anaphylaxis management plan were also predictors.
Jones et al. (2018)	England and Scotland, UK	21 participants (12–21 years), with a ‘range of allergies’, however, all in demographic table reported to have FA.	Telephone interviews analysed with thematic content analysis.	Support groups were believed to be useful as they were a place to share experiences, boost inclusivity, increase confidence and reduce feelings of isolation.
Macadam et al. (2012)	UK	20 participants (12–18 years) with ‘food or venom allergies.’	Interviews were thematically coded.	Themes: The type of allergy; role of circumstances; factors associated with device design; the responsibility and attitude of others; Teenager's feelings and concerns.
MacKenzie et al. (2010)	Isle of wight & portsmouth, UK	21 participants (13–18 years) with evidence of IgE‐mediated food hypersensitivity (FHS).’	Interviews analysed by the phenomenological method of Giorgi and Giorgi.	Themes: Living with FHS as a way of life/coming to know FHS as a way of life; living with FHS as experiencing and coping with burden; alleviation/exacerbation of the burden of living with FHS; living with FHS involves managing acceptable risk.
Marklund et al. (2007)	Stockholm, Sweden	17 participants (14–18 years), ‘exclusion diets due to food hypersensitivity.’	Three focus group interviews and six individual interviews analysed with qualitative content analysis.	Themes: Perceiving oneself as being particular; feeling constrained; experiencing others' ignorance; keeping control; feeling it's okay.
Monks et al. (2010)	Southampton, UK	18 participants (11–18 years), ‘teenagers with FA’, ‘recruited from clinic.’	Questionnaire (demographics and allergy management) and interviews analysed using a thematic approach.	Themes: Allergen avoidance; being prepared for reactions; treating reactions.
Saleh‐Langenberg et al. (2016)	The Netherlands	55 adolescents (13–17 years), ‘food‐allergic adolescents prescribed an AAI.’	Measures: FAQLQ‐TF, FAIM‐TF, IPQ & STAI. Analysis: Spearman's correlations, Fisher's exact test, Mann–Whitney *U*‐test.	Adolescents were (extremely) positive about AAIs. Those reporting a greater burden of treatment believed that they were less likely to be able to deal with a reaction successfully. Low burden of treatment was reported by adolescents who believed the AAI has an agreeable shape and gives a feeling of safety. High burden of treatment was associated with the belief AAI carriage was inconvenient.
Sommer et al. (2014)	Isle of Wight, Portsmouth and Southampton, UK	25 participants 7 with FA and 18 with no FA. (12–18 years). ‘Food‐allergic teenagers.’	One focus group with no FA (*n* = 11) and 14 interviews (7 with FA and 7 with no FA).	Themes: Variety and enjoyment of food as a learning process; body awareness, feelings and temptations of foods; parental control versus convenience; eating as social experience; routine, tradition and environment; knowledge shapes understanding of foods.
Stensgaard et al. (2017)	Denmark	5 families (adolescent participants 15–16 years), ‘adolescent with peanut allergy.’	Individual semi‐ structured interviews analysed with Ricoeur's theory of interpretation.	Themes: The nuclear family – safety and understanding; when the nuclear family is challenged; the importance of having a social life.
Stjerna (2015)	Sweden	10 participants, (11–17 years) from ‘with food allergies.’	Interviews analysed thematically.	Themes: management of health risks; management of social risks in different places.

Abbreviations: BIPQ, Brief Illness Perception Questionnaire; CS‐SRM, Common Sense Self‐Regulation Model; FAQLQ‐TF, Food Allergy Quality of Life Questionnaire: Teenager Form; FAIM‐TF, Food Allergy Independent Measure: Teenager Form; HBM, Health Belief Model; IPQ, Illness Perception Questionnaire; STAI, State‐Trait Anxiety Inventory.

### 3.2 Quality appraisal of studies

The MMAT^18^ was used to assess the quality of the studies included in the systematic review. Included studies were assessed by KN and reviewed by RK and no studies were deemed unsuitable for analysis due to poor quality. Multiple papers had arguably small sample sizes; however, these were included due to the limited studies on this topic and following recommendations by Braun and Clarke^20^ regarding sample sizes for qualitative studies. Multiple studies with the same author also included similar numbers of participants and inclusion criteria, however, as it is not clearly stated whether the samples were the same or different, and there were some different findings reported, all were included for analysis. Full quality appraisal can be found in Table 3.

**TABLE 3:** Adapted mixed methods appraisal tool

Category	Question	Yes	No	Can't tell	Comments
Screening (All papers both qualitative and quantitative)	S1. Are there clear research questions?	All			
	S2. Do the collected data allow to address the research questions?	All			
Qualitative: Akeson et al. (2007), Dean et al. (2016), DunnGalvin et al. (2009), DunnGalvin et al. (2021), Fenton et al. (2011), Fenton et al. (2013), Gallagher et al. (2011), Gallagher et al. (2012), Jones et al. (2018), Macadam et al., 2012), Mackenzie et al. (2010), Marklund et al. (2007), Monks et al. (2010), Sommer et al. (2014), Stensgaard et al. (2017), Stjerna (2015)	1.1. Is the qualitative approach appropriate to answer the research question?	All			
	1.2. Are the qualitative data collection methods adequate to address the research question?	All			Macadam et al. (2007) uses both interviews and focus groups yet analyses them in the same way without discussion of how data from the two data collection methods differ.Monks et al. (2010) use a ‘thematic approach’ to analyse their data but do not determine how the data is analysed for example, thematic analysis (Braun and Clarke).
	1.3. Are the findings adequately derived from the data?	All			Akeson et al. (2007) has 15 participants, and Stjerna (2015) recruited 10 participants, which may be perceived as a small sample.
	1.4. Is the interpretation of results sufficiently substantiated by data?	All			
	1.5. Is there coherence between qualitative data sources, collection, analysis and interpretation?	All			MacKenzie et al. (2010) participants listed as having food hypersensitivity (FHS) yet have been diagnosed as IgE‐mediated in an allergy clinic by skin‐prick testing, positive food challenge or serum‐specific IgE results. This different term does not seem to affect results.
					Dean et al. (2016), Fenton et al. (2011) and Fenton et al. (2013) have the same N of participants and inclusion criteria, and findings are reported by the same group of authors. Similarly, the studies by Gallagher et al. (2011 & 2012) have the same N and inclusion criteria.
Quantitative descriptive: James et al. (2020) Jones et al. (2014) Jones et al. (2015) Saleh‐Langenberg et al. (2016)	4.1. Is the sampling strategy relevant to address the research question?	All			
	4.2. 4.2. Is the sample representative of the target population?	James et al. (2020), Jones et al. (2014), Jones et al. (2015)		Saleh‐Langenberg et al. (2016)	Saleh‐Langenberg et al. (2016) has 55 participants which is arguably low and participant demographics such as age and gender are not stated.
	4.3. Are the measurements appropriate?	All			
	4.4. Is the risk of nonresponse bias low?	James et al. (2020), Jones et al. (2014), Jones et al. (2015)	Saleh‐Langenberg et al. (2016)		Saleh‐Langenberg et. al (2016) non‐response bias cannot be determined as there is no indication of the number of participants who declined or did not respond.
	4.5. Is the statistical analysis appropriate to answer the research question?	Y			Jones et al. (2014 & 2015) have the same N of participants and inclusion criteria.

*Note*: Other paper versions were not included in this review. Definitions and guides for MMAT may be found at Hong et al. (2018).

### 3.3 Thematic analysis

Five themes were identified in the data: (1) navigating FA in different environments; (2) carriage and use of adrenaline auto‐injectors; (3) managing the risk of anaphylaxis; (4) beliefs and understanding of others; (5) food‐allergic identity.

#### 3.3.1 Navigating food allergy in different environments

Beliefs about differences in management in spaces such as friends' houses, school, restaurants and abroad on holidays were discussed by adolescents in *N* = 12 studies and were affected by perceived risk, predictability, familiarity and distance from external help^11^, ^21^, ^22^, ^23^ such as hospitals. The family home or where a parent was present was considered safest^21^, ^23^, ^24^, ^25^ as the adolescent could relax and leave responsibility of their safety to someone else.

Concerns of missing out were apparent in places difficult to manage FA. Restaurants and school trips were described as annoying as adolescents could not eat ‘the same as everyone else’.^26^ Scepticism of expertise of catering staff was an important factor in the eating out experience with concerns about food prepared in unknown places.^8^, ^25^ Asking staff about an allergen was embarrassing,^27^, ^28^ and difficult,^24^ especially where staff were believed to be indifferent.^10^, ^25^ These beliefs were also expressed on holidays abroad, dealing with new foods and a different culture. Adolescents preferred to eat foods they knew were safe or rely on parental judgment, especially if there was a language barrier.^23^

Schools were believed to be risky depending on perceived support and perceived efficiency of school policies. Secondary school was viewed as more dangerous than junior school,^28^ due to less organisation and control from teachers. Adolescents believed secondary school to be exclusionary, isolating them by making them sit alone at lunch or excluding them from school trips.^28^, ^29^ However, it is not clear whether this was due to teacher choice or school policy. Adolescents believed they must be cautious managing FA at school due to difficulty determining risk and lack of trust in teachers. Some did not feel safe due to concern of contamination or previous experience of bullying, including threats to contaminate food or having their allergen thrown at them.^24^, ^26^ Adolescents described instances where they left the classroom against teacher wishes because they did not feel safe.^29^ Teachers were seen as unable to help due to the adolescent with FA being a minority in the classroom.^28^ Adolescents believed there was greater potential risk of encountering allergens or a reaction due to a high volume of students, unsupervised lunch areas, common eating areas and untrained staff.^24^, ^28^

Where schools had attempted to be accommodating, such as using allergen‐free ingredients in food technology classes, adolescents felt safer and included.^21^ Others believed special treatment because of their FA highlighted them as different to their peers.^29^ Some adolescents believed avoidance was the best way to cope with this, finding safe spaces where food was not allowed^23^ and managing their stress by reducing their risk of having a reaction.

#### 3.3.2 Carriage and use of adrenaline auto‐injectors

Beliefs about AAIs were discussed in *N* = 10 studies and were associated with likelihood of the adolescent carrying their AAI at all times and considered situational factors such as location, distance from home or parents, possibility of the allergen being present and whether they had visited a place before.^8^, ^9^, ^10^, ^11^ Barriers to AAI carriage were believed to be inconvenience of the size and difficulty of use.^8^, ^9^ Some adolescents were also afraid to administer^26^ due to fear of the needle, even in the event of a reaction.^10^, ^11^

Those who strongly identified with their FA and had stronger feelings such as anger or anxiety were more likely to carry their AAI.^6^ Also, adolescents with higher perceived severity and susceptibility to having a reaction were more likely to carry their AAIs as they considered it a protective tool. A quantitative study examining the predictive ability of the Health Belief Model^6^ found that barriers such as being seen as different or AAI carriage being inconvenient resulted in lower adherence to AAI carriage. Adolescents who viewed their FA as unstable, unpredictable and episodic were also less likely to be adherent to self‐care behaviours. This may be due to infrequency of reactions, leading to the belief that carrying an AAI is unnecessary.^6^ Findings from Gallagher et al.^9^ in a qualitative study suggested that barriers such as fear and uncertainty of how to use the AAI and failure to recognise anaphylaxis may reduce AAI use.

When the perceived risk of reaction was low, such as going to play football, adolescents were less likely to carry their AAIs.^10^, ^22^ More boys reported being inconvenienced than girls by the size of AAIs, stating they were too large for a pocket^9^, ^10^, ^11^ and believed they would be more likely to carry the AAI if they were smaller.^10^ Some were embarrassed of their AAIs and less likely to carry them because of shame, fear of being seen as different or irritation at having to explain themselves. However, some adolescents acknowledged that the benefits outweighed the inconvenience.^9^ Participants reduced discomfort of AAI carriage by leaving it with a friend, teacher or in their bag nearby.^11^

Beliefs about how to use AAIs were also salient and discussed in six studies. Jones et al.^7^ found the majority of their sample believed they could correctly use their AAI with 40% feeling ‘sure’ and 37% feeling ‘absolutely sure’. This contrasts with the findings of qualitative papers with a similar age range (13–17 years) from Scotland, Europe, and the Netherlands, as well as another quantitative study in the Netherlands where adolescents did not believe they could use their AAIs properly and feared they would use it wrong.^9^, ^27^, ^30^ Saleh‐Langenberg et al.^30^ suggested that lack of confidence using AAIs can result in a higher perceived burden. As some adolescents with FA were unsure of how to use AAIs, this could lead to misfires or reluctance to use the device.^11^ Adolescents and their siblings were afraid of using the AAI and of contacting emergency services, preferring to use antihistamines due to their familiarity.^26^ Anxious beliefs around the needles resulted in preferring someone else to administer the AAI^9^, ^10^ or for the AAI not to be used at all.^11^

#### 3.3.3 Managing the risk of anaphylaxis

Adolescents believed it was difficult to manage the risk of an allergic reaction^25^ and these beliefs were discussed in *N* = 11 of the studies. Adolescents were either dismissive towards high‐risk behaviours that may potentially result in anaphylaxis or expressed a great fear of allergic reactions, which they associated with severe danger.^5^, ^8^, ^21^, ^25^, ^28^ Some adolescents presented both beliefs, being very aware of their FA yet simultaneously viewing it as ‘no big deal’.^22^

Where peers were all eating a food which potentially contained an allergen, this increased likelihood of consumption.^10^ Eating a potential allergen despite the risk was undertaken to ‘fit in’ and gain the trust of others, and to increase independence, control and empowerment.^24^ Some also believed that certain foods, such as chocolate, were worth the risk of having a reaction.^21^, ^25^

Some adolescents were more likely to eat food with a warning label if a parent was present compared to just friends, as they felt safer.^10^ However the majority of adolescents were dismissive of ‘may contain nut’ warnings unless the product was made in a factory that made peanut products specifically.^10^ Adolescents were also sceptical towards the legitimacy of the presence of allergens in foods with ‘may contain’ labels and the process of constant checking was believed to be annoying^8^ and restrict their food choices. They believed less use of these ‘defensive’ warnings where risk was low as well as simpler and more consistent allergy warnings could improve allergen avoidance^10^ as labels may be taken more seriously.

#### 3.3.4 Behaviour and understanding of others

Beliefs towards FA from others such as friends, classmates, school staff, parents and the wider community were discussed in *N* = 14 of the studies. Adolescents in *N* = 8 of the studies said that once their FA was disclosed to their classmates, they experienced discrimination and classmates being mean‐spirited.^29^ Some expressed frustration that peers tended to focus on the limitations of their FA, identifying them as different and making the situation difficult to cope with.^21^ To avoid being excluded, some adolescents purposely did not disclose their FA, only confiding in close friends,^5^, ^27^ believing others would not understand. Classmates were highlighted as needing education and information to increase understanding.^10^ Jones, Sommereux and Smith^31^ emphasised the importance of social support groups. Being able to speak with others of a similar age and similar experiences with FA was believed to be helpful as they felt understood, which increased confidence.

A lack of education and awareness of others was the greatest barrier to adolescents with FA being understood, accepted and becoming independent.^28^ Adolescents also believed they could not necessarily trust adults due to a lack of understanding of FA and felt they needed to take control themselves to prevent a reaction. This lack of awareness around FA, for example, thinking an allergic reaction could just cause a rash rather than be life‐threatening,^5^, ^21^ led to adolescents feeling uncertain about how to manage different situations such as ordering food.^8^ Adolescents felt frustrated and believed their FA was not taken seriously by others and often ignored in schools by teachers and peers^21^, ^25^ and even in situations where allergen information is important such as restaurants.

Parental beliefs and behaviour may also affect adolescent beliefs and were discussed in *N* = 8 studies. Some parents highlighted the risks, encouraging hypervigilance and reminding of possible consequences, which sometimes led to conflict.^5^, ^24^, ^32^ Other adolescents accepted it was their parents showing that they cared and felt safer.^32^ Stengaard et al.’s^26^ study highlighted that where parents were divorced, adolescents believed it was the responsibility of the parent to educate new members of the family. In cases where adolescents felt unsupported by parents, this led to less trust and anxiety at home. Further conflict may result in a breakdown of routine when with grandparents, who were described as more lenient.^24^ When there was a family member or friend who also had a FA, adolescents felt more accepted^11^, ^21^ as otherwise some family members struggled to empathise.

#### 3.3.5 Food‐allergic identity

A total of *N* = 17 studies discussed beliefs related to identity and FA. Some adolescents believed FA was difficult as it was believed to be a big part of their identity, that they were powerless, and their FA was unmanageable. In response, maladaptive coping strategies including isolating themselves or excessive hand washing were reported.^28^ High illness concern and emotional representation, especially in females may lead to emotional difficulties due to their FA and would require further support.^33^ Believing it was necessary to depend on others and feeling a lack of control in relation to their own bodies was difficult for some adolescents,^21^ especially at an age where their peers were becoming more independent. Participants in Stjerna's^21^ study were concerned about causing burden to others by restricting them and forcing them to adapt because of their FA, and that this may lead to the adolescents with FA being excluded. Those who engaged in risky behaviour, such as eating food which may contain allergens, felt less fear and had a stronger sense of self with increased confidence.^8^, ^24^ For these adolescents, anaphylaxis was considered the same as any other risk such as crossing the road.

Others expressed acceptance of their FA but felt it should not define them. They adopted optimistic beliefs that it ‘could be worse’, that others had worse experiences, or their FA had improved as they aged.^8^, ^25^ Some were hopeful they may outgrow their FA and that it would be a temporary issue rather than a life‐long burden,^8^ however James and Caballero^33^ suggest the belief of FA being a life‐long condition increased between 14 and 18 years of age. Differences in beliefs may be attributed to different ages, suggesting this may impact beliefs about identity. After having FA for some time, some adolescents grew accustomed to having FA and expressed acceptance and resignation^24^ and looked to balance risk, so the FA did not dominate their lives.^9^, ^34^ In MacKenzie et al.’s^8^ sample aged between 13 and 18 years, they learned to adapt to having FA while younger participants were more frustrated by barriers from their FA. In contrast, Dean et al.’s^29^ sample showed that younger participants (8–12 years) were more relaxed and considered FA a ‘diet’ whereas older participants (13–17 years) reported that it was ‘a big deal’ and considered life or death. Fenton et al.^28^ supports the shift in attitudes across age as reporting stronger feelings of safety in elementary school due to parents being more present, supervision from trained staff and a consistent routine.

Considering the future, gaining choice and control led to empowerment and gaining trust in themselves and their environment, resulting in a more positive outlook.^28^ When reflecting on a future away from parental safety, adolescents believed although they would never be completely safe, they would get increasingly better at FA management^21^ and in their own safe space would have control over what food they were exposed to.^25^

### 3.4 Subgroup analysis

Subgroup analysis was explored through results based on age, gender, geographical location, method and allergen to determine whether this may influence results. Gender differences between participants were limited, however frustration with AAI carriage was reported as more substantial in male participants.^9^, ^10^, ^11^, ^32^ Studies did not explicitly discuss differences in beliefs dependent on allergen.

Methodologically, there appeared to be minimal contrasting findings apart from where Jones et al.^7^ found the majority of their sample believed they could correctly use their AAI. This contrasts with the findings of qualitative papers and one other quantitative paper with a similar age range (13–17 years), where adolescents did not believe they could use their AAIs properly and feared they would use it wrong.^9^, ^27^, ^30^ However this may be due to differences in the location the samples were sourced (South East‐England for Jones et al.^7^, and contrasting studies including samples from the Netherlands, Scotland and multiple locations in Europe^9^, ^27^, ^30^).

There were more beliefs around anaphylaxis and FA as ‘a way of life’ in UK samples^8^, ^22^ compared to increased fear for safety, especially in relation to severe reactions, in Swedish samples^21^, ^25^ and 13–17 year olds of Dean et al.’s^29^ Ontario sample, who viewed FA as a ‘big deal’. This difference may be attributed to a shift across time, as the more anxious beliefs are displayed in more recent studies, which may be linked to an increase in FA prevalence. Another factor that may have influenced Dean et al.’s^29^ sample was Sabrina's Law, which was enacted after the death of a 13‐year‐old girl in Ontario, where this study was conducted. Fenton et al.^28^ is also based in Canada yet focuses more on coping with illustrative methods while Dean et al.^29^ considers stigma with more typical qualitative interview methods. DunnGalvin et al.^5^ explicitly discussed a period of transition around 12 years of age, where increased conflict with parents was reported. Conflict with parents was also reported in Fenton et al.^24^ and Gallagher et al.^9^ though the specific age of this conflict emergence was not discussed. DunnGalvin et al.^27^ was the only study to include multiple countries and highlighted the differences as bullying not reported in Denmark and the Netherlands, and embarrassment not reported by adolescents in Spain, Italy, or the Netherlands. Cultural differences may be related to various factors such as parenting styles, education, healthcare systems and policies, although future research would be required to explore these factors.

## 4 DISCUSSION

This systematic review has uniquely identified five themes regarding beliefs about adolescent FA; 1) navigating FA in different environments, 2) carriage and use of AAIs, 3) managing the risk of anaphylaxis, 4) behaviour and understanding of others, and 5) food‐allergic identity.

Schools were believed to be risky places depending on how supported adolescents felt, and secondary school was viewed as more dangerous.^27^ Stigma and bullying are issues in need of attention as not understanding the severity of FA can lead to fatalities, as in the case of West London student Karanbir Cheema who died of a severe allergic reaction aged 13 years.^33^ Concerns around exclusion in schools from studies in this review have also been reported in other research looking into FA policies in schools.^35^ Educating those with FA about risk rather than allowing fear to be a barrier may improve beliefs towards safety of places. Teaching adolescents how to manage risky situations such as navigating restaurant menu ingredients or eating with friends may also increase independence.

AAIs were highlighted as a big concern in this review, with subgroup analysis identifying more frustration with AAIs in male participants. Carrying their AAI despite its perceived inconvenience could be attributed to increased perceived severity of FA. Leach, Smith, Brown, Davies and Jones^36^ suggest that adolescents aged 13–18 years were conscious about safety, speed of administration, accessibility and carriage, comprehensive instructions, indication of correct administration, visibility and precise drug delivery. AAI size and needle concern were also important, a re‐evaluation of design to make the AAI smaller may increase likelihood of carriage.^10^ Reassurance about the needle with frequent training and provision of trainer pens from allergy clinics may also increase confidence on administration and reduce apprehension, reducing mistakes such as not holding the device in place long enough.^37^ Good administration was seen more in those who had a history of anaphylaxis, were over 18 years of age, prescribed an AAI more than 30 months, membership of a support group and AAI administration training by an allergist, highlighting a need for support in those under 18 years of age.

When managing the risk of anaphylaxis, adolescents found it difficult to balance increasing independence and accepting more responsibility for their FA, which is experienced uniquely in adolescence. Beliefs seen in this review were also seen in an older sample in Greenhawt et al.’s^38^ study of college students, with 60.3% (173 students) not always avoiding known allergens due to no previous serious reaction (37.6%) or the perception that it was not a risky behaviour (20.8%). A shift in beliefs and behaviour may begin in adolescence as suggested by DunnGalvin et al.,^5^ leading to the potential for further risky behaviour in early adulthood. More research into how beliefs and attitudes change throughout development, such as moving to further education would be beneficial to give insight into how to prepare for these challenges, with consideration of cultural differences as identified in the subgroup analysis. Furthermore, focus of clinics on facilitating healthy behaviours in adolescence and efficiently preparing young people for adult life may be protective against this rise in maladaptive behaviours.

A common risky behaviour was dismissal of ‘may contain’ food warnings and consuming foods that may not be safe, in line with findings from Greenhawt et al. and Sampson et al.^4^, ^38^ This is a concern as intentional exposure to allergens in children leading to an allergic reaction were cited as due to not yet having a serious reaction.^39^ A study of food choices in nut‐allergic consumers,^40^ highlighted three strategies: past experiences of food consumption; sensory factors to determine risk; and quality of the product or the place the food originated from. Adolescents in this review also discussed using past experiences, supporting this technique. However, they also were influenced by parents or friends.

The visual aspect of the allergen labels such as where they were located on the packaging was is seen as important^41^ as increased visibility may reduce accidental ingestion. Where possible, greater information about food production, regulations and the labelling process from food businesses and food guidelines may encourage adolescents to take labels seriously and reduce scepticism. This may assist with the prioritisation of confident food choices as a protective measure for people with FA, as highlighted in a recent survey by the Food Standards Agency.^42^ With the introduction of oral immunotherapy treatments (OIT) for FA,^43^ it is important to balance beliefs and understanding that while we are making great strides in FA treatment, we do not yet have a cure. OIT has been found to be making great improvements in quality of life in adolescents with FA, leading to greater dietary choice, reduced anxiety, and increased social inclusion. However, OIT also causes anxiety in paediatric patients due to fears of needles and reactions,^44^ as found in this review regarding beliefs of needles in AAIs, and so understanding of beliefs is important to support treatment.

When discussing behaviour and understanding of others, peer relationships were thought to be difficult to manage due to the lack of knowledge of FA among peers, supporting research that peer attitudes are important for adolescents with allergic conditions^13^ leading adolescents to be more reluctant to disclose their FA.^45^ Adolescence is a sensitive time for social relationships and personal development. Lack of support from peers during this time could result in negative emotions and reduced quality of life in those with FA, and lead to greater distress in parents.^25^, ^46^, ^47^ Jones et al.^30^ highlighted the importance of peer support groups in those with FA, as this led to increased confidence and feeling understood. Allergy clinics should work in collaboration with existing support groups and encourage creation in areas where none are yet established to foster these support systems. Changing beliefs and behaviours of peers through education of FA and FA guidelines through incorporating tools such as a whole school awareness toolkit^48^ may improve beliefs and behaviours of those with FA and reduce risky behaviour. Educating the wider community, beyond immediate peers, is also important for decreasing risk of reactions and reducing the negative impact of diagnosis and FA management.^49^ Clinicians should also be aware that socioeconomic and racial factors may affect FA knowledge and should offer further support accordingly.^50^

In family relationships, parental support and conflicts with independence were common in adolescents with FA and despite most participants having a good relationship with their parents, some felt their parents wanted to ‘control their lives’.^25^ This is supported by recent research from McLaughlin, Humiston and Peterson^51^ who found that as parental worry increased, so did limitations on children regarding FA‐related social activities. Previous research by Van der Velde et al.^52^ also suggests conflict, and that increased perceived severity of FA and poorer illness comprehension is linked to adolescent‐parent conflict in Dutch adolescents. The increased independence seen in adolescents in this study may be due to cultural differences as suggested in the subgroup analysis, as the study was conducted in Denmark which may have different parenting styles to the other studies, however more cross‐cultural data would be required to draw any conclusions. Psychological support to manage this time of tension in family relationships may improve adolescent behaviour and family mental health, with Stensgaard et al.^53^ highlighting the benefit of allergy clinics in supporting parents and McLaughlin et al.^51^ suggesting that increasing parent self‐efficacy may help with reducing worry and social restriction of children with FA. Anxiety in adolescents with FA and parents^54^, ^55^, as also shown by findings from this review, is a key area for clinicians, which may be supported by appropriate understanding of beliefs.

In discussing FA identity, an increase in normalisation was desired and may be reached through education around FA and FA management. As adults with FA can still experience anxiety and struggle with management^56^, as adolescents were seen to in papers in this review, it is important to support the younger generation of adolescents with FA to reduce this. Greater understanding from healthcare professionals about the importance of beliefs, how they relate to behaviour, and the necessity for increased information in all areas from schools to restaurants and the wider community is important to support adolescents with FA, with specific mental health advice and management strategies necessary in allergy clinics. Further education on managing risks, encouraging independence and improving beliefs in adolescents' capability and perceived control could be beneficial, reduce anxiety surrounding allergic reactions and lead to more positive attitudes.

Adolescents in all studies expressed that education is important for moving forward. The need for further research to support independent self‐management of FA has been requested by adolescents^9^ in addition to more supportive peers and healthcare professionals listening to them without judgement.

## 5 STRENGTHS

The main strength of this review was the identification of themes that were not present in the original papers but appeared after synthesising results across studies, offering novel findings that have not been previously investigated in this age range, as individual papers focused on more specific topics (e.g., AAIs). A further strength of this review was the inclusion of both qualitative and quantitative research of various methodologies, offering different perspectives which has not been explored previously. Quantitative studies provided larger sample sizes, whereas qualitative papers had more depth and offered possible explanations for the quantitative results.

The findings of this review are supported by the results from papers using various methodologies, identifying similar factors regardless of methodological differences. This systematic literature review also considers the effectiveness of papers that included theoretical models^6^, ^7^ compared to those that did not and found in particular the mention of barriers to be a key component of adolescent behaviour, as well as severity, which is considered in qualitative studies where adolescents feel peers do not understand the seriousness of FA as a condition.

## 6 LIMITATIONS

Many of the papers included did not primarily aim to explore beliefs regarding FA, resulting in data having to be extracted and interpreted. The review was further limited by the information (quotes or data) included in the original papers as the original transcripts or data sets were not available. With the open science movement gaining traction, these resources may be available to future reviewers to collect data that may otherwise be missed. Grey literature and unpublished studies were also outside of inclusion criteria and the open science movement may make these materials, as well as data such as original transcripts, more accessible.

Included papers had some limitations which may impact the quality of the research, with the majority of studies having very small sample sizes; four studies featuring 10 or less relevant participants,^8^, ^21^, ^22^, ^34^ and Saleh‐Langenberg et al.’s^30^ study also had a small sample size for quantitative analysis, with 55 participants who were not demographically defined. Non‐response bias was also not reported. The majority of samples were also from White/Western European countries, with little consideration to ethnicity or socio‐economic status. This limits the scope and generalisability to other cultures and racial and ethnic groups, highlighting how little this construct has been studied with a need for culturally appropriate replication.

## 7 IMPLICATIONS AND FUTURE DIRECTIONS

FA beliefs are affected by various factors including place, AAIs, risk, peers and identity. As a lack of trust was reported in secondary schools, especially in teachers,^28^ it would be useful to develop educational and peer‐support interventions for 11–19‐year olds, during a time when they experience transition into and out of secondary school, especially as a shift is suggested at around 12 years of age, where children rely less on parents and teachers and are less likely to disclose their FA to friends.^5^, ^45^ Teachers and other school staff may benefit from training regarding managing FA and how to navigate risk. Peers in school should receive further education about FA, potentially utilising whole‐school toolkits such as by Higgs et al.^48^ which may reduce bullying and stigma.^24^, ^28^, ^29^ Adolescents feel peers could be more supportive with their allergic conditions,^12^, ^13^ and with increased peer acceptance and understanding, beliefs may change and shape attitudes towards FA, its impact and management. Ages of transition such as the move from primary to secondary school, and then the move to college or University would benefit from further research. These age groups as a focus are important as it is the age adolescents seek further independence and responsibility and increase control of managing their FA.

## 8 CONCLUSION

Greater understanding from healthcare professionals about the importance of beliefs and how they relate to behaviour and the necessity for increased information in all areas, from schools, restaurants, and the wider community, is essential. In clinics, psychological support for families, FA management in adolescents seeking independence, and addressing misinformed or maladaptive beliefs is essential. Anxiety around FA must be addressed in children, adolescents and parents so that FA becomes manageable in the transition to independence and adulthood. Regular training and support from clinics may improve protective behaviours such as carriage and use of AAIs and checking of food labels, though it is important to address beliefs developed from fear or misinformation. Adolescents in all studies expressed that further education is important for moving forward and improving peer understanding, which may help shape both attitudes and behaviour. With further research, and psychological, behavioural and clinical recommendations, adolescents with FA may have more confidence in managing their condition and progress with reduced risk of allergic reactions.

## CONFLICT OF INTEREST

Rebecca C. Knibb receives consultancy fees from Aimmune. Kristina L. Newman and Angel Chater have no conflicts of interest.

## AUTHOR CONTRIBUTIONS

**Kristina Newman:** Conceptualization, data curation, formal analysis, funding acquisition; supporting, investigation, methodology, project administration, resources, software, supervision, validation, visualization, writing – original draft, writing – review & editing. **Angel Chater:** Writing – review & editing. **Rebecca Knibb:** Conceptualization, data curation, formal analysis, funding acquisition, investigation, methodology, project administration, resources, software, supervision, validation, visualization; supporting, writing – original draft, writing – review & editing.
